# Mapping and monitoring carbon stocks with satellite observations: a comparison of methods

**DOI:** 10.1186/1750-0680-4-2

**Published:** 2009-03-25

**Authors:** Scott J Goetz, Alessandro Baccini, Nadine T Laporte, Tracy Johns, Wayne Walker, Josef Kellndorfer, Richard A Houghton, Mindy Sun

**Affiliations:** 1 Woods Hole Research Center, 149 Woods Hole Road, Falmouth, MA 02540, USA

## Abstract

Mapping and monitoring carbon stocks in forested regions of the world, particularly the tropics, has attracted a great deal of attention in recent years as deforestation and forest degradation account for up to 30% of anthropogenic carbon emissions, and are now included in climate change negotiations. We review the potential for satellites to measure carbon stocks, specifically aboveground biomass (AGB), and provide an overview of a range of approaches that have been developed and used to map AGB across a diverse set of conditions and geographic areas. We provide a summary of types of remote sensing measurements relevant to mapping AGB, and assess the relative merits and limitations of each. We then provide an overview of traditional techniques of mapping AGB based on ascribing field measurements to vegetation or land cover type classes, and describe the merits and limitations of those relative to recent data mining algorithms used in the context of an approach based on direct utilization of remote sensing measurements, whether optical or lidar reflectance, or radar backscatter. We conclude that while satellite remote sensing has often been discounted as inadequate for the task, attempts to map AGB without satellite imagery are insufficient. Moreover, the direct remote sensing approach provided more coherent maps of AGB relative to traditional approaches. We demonstrate this with a case study focused on continental Africa and discuss the work in the context of reducing uncertainty for carbon monitoring and markets.

## Background

The monitoring requirements for reducing emissions from deforestation and forest degradation have been widely discussed and documented in a range of publications, including overviews of the general requirements to meet policy needs [1] as well as a variety of papers on the technical aspects and limitations of various monitoring approaches [2-5]. The general consensus of these documents is that monitoring of forest cover change using satellite remote sensing is practical and feasible for determining baseline deforestation rates against which future rates of change can be based, provided that adequate validation and accuracy assessments are conducted and documented. The type of monitoring and baseline approach used has been the subject of much discussion, with a range of modifications proposed to deal with equity issues among countries with different historical rates of deforestation. Methods to map and monitor forest degradation, in which only a portion of the forest stock is removed, have also been developed. These range from straightforward visual interpretation of satellite imagery at multiple spatial scales (grain sizes) [6] to semi-automated algorithmic techniques that require technical expertise to implement [7]. Mapping and monitoring of carbon stocks, on the other hand, has often been regarded as beyond the current capability of satellite remote sensing technology, despite great need [8], partly because much of the research on this topic has historically focused on field sampling approaches [9]. Nonetheless, mapping carbon stocks over large areas without satellite data is clearly problematic [10].

Basing UNFCCC (United Nations Framework Convention on Climate Change) REDD (Reduced Emissions from Deforestation and Degradation) policies on a carbon stock mapping approach would have a number of benefits relative to approaches based solely on field sampling and forest inventories. This is true not only in terms of improving estimates of carbon stored in forests for the emerging carbon markets, by providing spatially explicit information on the location of carbon stocks, but also with respect to avoiding the ambiguities, uncertainties and outright differences among land cover type classifications [11]. A carbon stock approach could allow countries to report at a higher IPCC (Intergovernmental Panel on Climate Change) reporting tier by providing country-specific data and advanced methods and data for land conversions, even at a Tier 3 level which is defined in the Good Practice Guidance (GPG) as including "models and inventory measurement systems tailored to address national circumstances, repeated over time, and driven by high-resolution activity data and disaggregated at sub-national to fine grid scales (GPG 3.17)" [12]. A carbon stock monitoring approach is directly linked with biomass dynamics. Since such an approach does not depend upon the determination of land use/cover types as a step in estimating biomass, the uncertainty associated with these classifications is removed. Land classifications may still be applied to the biomass map for the purposes of accounting and reporting, but they are no longer a necessary step in the determination of the biomass of the land area. A carbon stock monitoring approach will introduce different, and perhaps additional, sources of uncertainty than other more traditional methods, but can reduce the overall uncertainty level below Tier 1 methods, and depending on the specific situation, is also likely to reduce uncertainties below Tier 2 methods, in addition to providing geographically explicit information on changes in carbon stocks. This approach could be used to obtain estimates for above-ground biomass (AGB) in all of the categories of LULUCF (Land Use, Land Use Change and Forestry) reporting, including categories where land classification remains the same (i.e. Forest Land Remaining Forest Land) and in categories defining changes in land use (i.e. Land Converted to Forest Land). Additionally, this approach would also allow a Tier 2 key category analysis (GPG 5.30), as it can provide specific uncertainty estimates for each category measured with this approach.

In the remainder of this overview we use carbon stock and above-ground biomass terminology interchangeably (biomass is typically 50% carbon). We recognize that carbon stocks can refer to below-ground and soil carbon as well, neither of which are directly discussed here.

## Overview of Satellite Measurements useful for Carbon Stock Mapping

### Synthetic Aperture Radar (SAR)

Since the 1960's, SAR has been used to produce images of earth-surface features based on the principles of radio detection and ranging (RADAR, often used as a synonym for SAR) and has been widely used to map AGB [13,14]. SAR systems are active, which means they transmit microwave energy and measure the amount of that energy reflected back to the sensor. As a result, SAR sensors can operate day or night while penetrating through haze, smoke, and clouds. The microwave energy transmitted by a SAR also penetrates into forest canopies, with the amount of backscattered energy largely dependent on the size and orientation of canopy structural elements, such as leaves, branches and stems. The sensitivity of SAR sensors to different AGB components is a function of the wavelength of the sensor, with shorter-wavelengths (X and C band) being sensitive to smaller canopy elements (leaves and small branches) and longer wavelengths (L and P band) sensitive to large branches and stems. Measuring the orientation (polarization) of the transmitted and received electro-magnetic waves allows for further sensitivity to AGB measurements. Also, the application of interferometric SAR (InSAR) is employed to improve retrievals of AGB through proxies of vegetation height [15]. Extensive analyses with existing SAR sensors, mostly L-band, suggest the sensitivity of radar backscatter "saturates" around 100 – 150 tons ha^-1 ^[13]. A number of radar satellites are currently in operation, including the Canadian RADARSAT 1/2 (C-band), the Japanese ALOS/PALSAR (L-band), the European ENVISAT/ASAR (C-band), the German TerraSAR-X (X-band), and the Italian Cosmo/SkyMed (X-Band). Several others are planned for launch within 5–10 years, including the ALOS follow-on mission (L-Band), the NASA DESDynI (L-band), the European BIOMASS (P-band), the German Tandem-X (X-band InSAR), and the German/Brazilian MAPSAR (L-Band).

### Light Detection and Ranging (lidar)

Like radar, lidar is based on the concept of actively sensing the vegetation using a pulse of energy, in this case from a laser operating at optical wavelengths (rather than at radio wavelengths). Lidar does not penetrate clouds but has the unique capability of measuring the three-dimensional vertical structure of vegetation in great detail, sometimes with hundreds of measurements in the vertical dimension for each location on the Earth [16]. Whereas lidar has only been widely used for a little more than a decade, primarily for forestry operations using aircraft-based sensors, it has revolutionized the way vegetation, particularly biomass, is measured from satellites [17-21]. There are few lidar instruments currently operating from satellite platforms, and none that were designed specifically for vegetation characterization. At least one such mission, a lidar on DESDynI, is planned for launch in the next few years. A currently operating satellite lidar sensor originally designed for monitoring ice dynamics, the Geoscience Laser Altimetry System (GLAS) onboard ICESAT, is being used for vegetation analysis despite having limited spatial coverage and a relatively large (ca 70 m) ground footprint.

### Optical

Optical remote sensing, i.e., passive sensing of visible and near-infrared reflectance from the earth, forms the basis for much of current global scale mapping (GoogleEarth, for example, is based on a combination of observations from the Landsat and Quickbird series of satellites). Optical measurements have been widely used in studies that link AGB measurements from the field to satellite observations, based on sensitivity of the optical reflectance to variations in canopy structure, but these have not proven to be consistent over large areas because surface conditions may change more rapidly than the repeat time of the cloud-free satellite observations, producing artefacts in the derived maps. This has been overcome using frequent repeat measurements from sensors such as the Moderate Resolution Imaging Sensors (MODIS) onboard the AQUA and TERRA satellites [20,22]. Despite some issues with the continuity of optical satellite missions [23] a wide range of sensors are expected to be operational well into the future.

### Multi-sensor Synergy

No single sensor on any satellite mission, whether radar, lidar or optical, can be expected to provide consistently infallible estimates of biomass, but use of these measurements in a synergistic fashion can potentially overcome the limitations of each (whether radar saturation, lidar sampling modes, or optical temporal mismatches). Moreover, some remote sensing observations may be more sensitive to AGB in specific environments (e.g. optical radiometry in more arid areas) or in areas with different AGB densities (e.g. lidar in dense humid forest).

## Comparison of Methods to Map Carbon Stocks

A number of approaches have been developed to map carbon stocks and AGB from the satellite observations described above. Each of the approaches relies on calibrating the satellite measurements to in situ estimates of AGB at field study plots. AGB is often determined using a combination of well documented allometric relationships between simple plot-level measurements (e.g. stem diameter, density and sometimes canopy height and/or depth) and AGB, where the latter is determined from trees that have been dissected, oven-dried and weighed [9,24,25]. This type of allometry has a long history and is used in daily forestry operations worldwide, although refinements are always needed and ongoing. We cannot describe all of the possible approaches to estimating AGB from satellite observations which include, for example, spatial interpolation and kriging of field measurements, but focus instead here on the most commonly used.

### Stratify & Multiply (SM) Approach

The simplest approach to derive carbon stock maps is to assign a single value (or a range of values) to each of a number of land cover, vegetation type, or other thematic map classes that have been derived from satellite data (or other map sources) and placed into categories (such as Evergreen Lowland Forest, Deciduous Forest, and the like). These thematic class areas are then multiplied by the assigned values to estimate total carbon stock values. Land cover maps are widely available from a number of sources, with the most consistent and best-documented effort to date being the Global Land Cover 2000 maps produced by a broad consortium of research groups [26]. This "stratify & multiply" approach is limited in a number of ways, but primarily by the wide range of AGB variability within any given thematic type class, and by ambiguities in the definition of those type classes (which is difficult to make universal and thus has consumed the better part of many workshops over the years).

### Combine & Assign (CA) Approach

An extension of the stratify & multiply approach is a "combine & assign" approach, which essentially makes use of a wider range of data sets and spatial information to extend the field AGB estimates. For example, population estimates (or maps derived from interpolating population location data) can be used together with vegetation type classes and any of a number of other spatial data layers in a geographic information system (GIS) to provide finer-grained units over which the field data can be applied (given that adequate field data exist to characterize each of the basic map units). A substantial advantage to this approach, besides finer spatial units of aggregation, is that different weights can be applied to various data layers in order to capture information that is known (such as locations where forests are more degraded around settled areas) or to average gradients across large areas (such as variations in vegetation density within type classes). Another advantage of this type of simplified GIS "modelling" is that values can be aggregated and provided for specific political jurisdictions [27] (see Figure). Despite these advantages, the combine & assign approach suffers from some of the same limitations as the stratify & multiply approach, particularly in that a representative value (or range of values) is assigned to, and assumed to be representative of, a given spatial unit and field data may not be available to adequately characterize those units. Moreover, it becomes increasingly difficult to acquire spatially consistent information (i.e., spatial data layers) needed for a combine and assign approach as the size of the study area increases.

### Direct Remote Sensing (DR) Approach

A more spatially consistent way to produce carbon stock maps is to extend the satellite measurements directly to maps by calibrating them to field estimates of AGB using any of a number of statistical or so-called "machine learning" techniques, such as neural networks or regression trees [28,29]. In the simplest terms this approach makes use of a set of field measurements to "train" an algorithm to develop a set of rules by which any combination of satellite observations (whether radar, lidar, optical, or a combination of these) produce a unique solution in terms of "observed" (i.e., field estimated) AGB. The approach is typically done in an iterative manner, repeatedly passing through the data sets to produce an optimized set of rules that account for the greatest amount of variability in the training data and, by so doing, produce the smallest error in the satellite-derived estimates of AGB. For example, maps have been produced at 1 km resolution using MODIS imagery across all of Africa, a continent of particular importance in the global carbon cycle [30], and validated using independent lidar data sets [20] (see Figure). Related analyses have been done for the Amazon basin [31], Russia [32] and the United States [33] using a similar approach. Multi-sensor synergy has also been used with a network of forest inventory data to produce ca. 1 hectare resolution biomass maps for tropical Costa Rica (see [11]) and is in progress for the conterminous U.S. [34]. Once the optimized rules are established for the training data, they are then applied to the satellite images to produce wall-to-wall maps with continuous values of AGB for each cell (pixel) of the image (or map). A key advantage of this approach is that the rules, once established, are easy to understand and can potentially be adapted to a monitoring framework (next section).

### Comparisons and Potential for Monitoring

**Figure 1:**
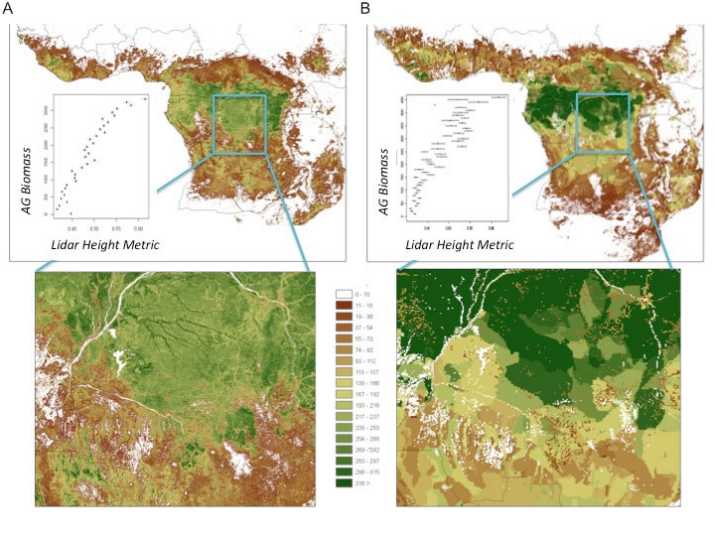
**Comparison of Central Africa Biomass Maps**. Map of above ground biomass (AGB) across Africa produced using a "Direct Remote Sensing" approach (A) [20] and a "Combine and Assign" approach (B) [27]. The top images show maps of AGB for the tropical forest regions of Africa, with boxes indicating those areas shown in the bottom images. The inset line graphs in the top images show how the range of AGB relates to independent lidar metrics that are closely related to field estimates of AGB. Less variability in the lidar height metric for each associated AGB value in the maps indicates lower uncertainty and error.

In addition to the general principles of the approaches, as described above, details of the data sets and specifics of the approaches used to produce the maps shown in Figure 1 are provided in the associated publications describing those maps [20,27]. Both approaches made use of similar allometric equations [9,24] (see 20 for more on this) relating the field measurements to estimates of AGB, but differ in the way those field data are associated with the GIS data layers (CA approach) or the satellite observations (DR approach). Comparing the maps visually shows the DR approach (Figure 1A) has more spatial detail across the region than the CA approach (Figure 1B), with both characterized by transitions between vegetation types ranging from dense humid forest in the Congo Basin to more open woodlands to the south. Note the more continuous nature of the DR map, and the more aggregated spatial units in the CA map. The comparisons to independent GLAS lidar metrics, which are closely related to field estimates of AGB [11,17,20] show a narrower range of variability within each AGB value in the DR approach, indicating less uncertainty at any given location in the DR map.

Even though a land cover map was not used (not needed) for the DR approach to estimate AGB, further comparison of the maps in Figure 1 was done by summarizing the AGB values in both maps by land cover type (Table 1). Although most of the classes show a similar average AGB values within each class of the Global Land Cover 2000 product [26], note how the averages based on the CA map tend to be higher than those derived using the DR approach (88% of the 25 cases). It is possible that the DR approach is systematically underestimating biomass within cover types, but validation of this map [20] and similar map products [29,31-33] show that not to be the case. The apparent bias in the CA approach is most likely associated with difficulties in assigning field plot measurements to more generalized land cover categories, as described earlier. This is most evident in classes that are often modified by human land use (e.g. evergreen lowland forest classes), but also in areas with little or no vegetation cover (see last 5 classes of Table 1). Note also that the CA approach indicates higher AGB in deciduous woodland than in closed deciduous forest, and other apparent irregularities (e.g. between shrublands and grasslands).

**Table 1:** Biomass Densities by Land Cover Type

Class Name	DR	CA	Δ
Closed evergreen lowland forest	216.3	273.5	57.2
Degraded evergreen lowland forest	121.2	171.5	50.3
Submontane forest (900 – 1500 m)	238.2	186.8	-51.4
Montane forest (>1500 m)	169.6	94.6	-75.0
Swamp forest	250.7	346.9	96.2
Mangrove	48.3	100.9	52.6
Mosaic Forest/Croplands	91.5	96.6	5.1
Mosaic Forest/Savanna	77.4	91.9	14.5
Closed deciduous forest	84.9	81.8	-3.1
Deciduous woodland	35.2	89.4	54.2
Deciduous shrubland with sparse trees	11.5	61.0	49.5
Open deciduous shrubland	12.8	61.6	48.8
Closed grassland	7.0	73.9	66.9
Open grassland with sparse shrubs	1.0	14.3	13.3
Open grassland	1.9	13.4	11.5
Sparse grassland	2.3	6.0	3.7
Swamp bushland and grassland	32.7	57.3	24.6
Croplands (>50%)	5.3	36.9	31.6
Croplands with woody shrubs	1.1	10.2	9.1
Irrigated croplands	1.6	44.8	43.2
Sandy desert and dunes	0.0	31.0	31.0
Stony desert	1.3	13.0	11.7
Bare rock	0.8	13.4	12.6
Salt hardpans	1.2	42.4	41.2
Water bodies	5.6	107.7	102.1

Land cover classes derived from the GLC2000, average AGB (tons ha-1) derived from the "direct remote sensing" (DR) and from the "combine and assign" (CA) approaches.

The column labelled Δ indicates the difference between approaches (CA-DR).

Any of the approaches described herein could potentially be used in a framework for monitoring AGB stock changes. An SM approach would use new land cover maps to estimate changes in stocks, but the maps would need to be recreated at both (each) time step at accuracies that exceed those of typical current map products (particularly given that errors multiply whenever categorical maps are differenced for two time periods). A CA approach could make use of successive field surveys to update maps derived using GIS models, but field data are unlikely to ever be sampled adequately for monitoring purposes, particularly outside "intact" forests [35], and the issues noted above with respect to use of progressive land cover maps (and other spatial data sets) also apply. Moreover, both of these approaches are prone to missing changes at grain sizes finer than the thematic map units, including forest degradation and afforestation (regrowth) at time scales that are meaningful to the UNFCCC process. These issues are unlikely to impact the DR approach for monitoring purposes because land cover maps are not required, the spatial unit is typically much smaller than the other approaches (i.e. the pixel size), and the data sets used (satellite observations) are not only more current but also more consistent through time (via routine calibration efforts). Unlike land cover or land use maps, the satellite observations used in the DR approach are sensitive to changes at the pixel level, thus even fine-scale changes associated with degradation and afforestation can be detected [11]. Nonetheless, more work needs to be done with the DR approach in order to refine the process, reduce uncertainty (error), and better quantify the requirements (in terms of sample sizes and spatial extents) for field data sets needed to calibrate and validate the models. This topic is the focus of both applied research for the UNFCCC process and basic research designing the next generation of satellite missions that will provide the essential data sets needed for any monitoring system relevant to reducing emissions from deforestation and forest degradation.

## Conclusion

Well documented techniques and satellite data enable reliable mapping of carbon stocks over large areas. Although a number of relatively simple methods exist for assigning field estimates of AGB to categories defined by vegetation type classes or weighted data layers in geographic information system models, the most spatially consistent maps are produced using models that derive continuous values (e.g. between 0 and 500+ tons per hectare) from statistically optimized decision rules. The techniques and data sources described herein are published in the refereed scientific literature and are progressing rapidly as new "data mining" techniques are advanced [28] and improved satellite remote sensing data become available [11]. This situation will improve further as new satellite missions come online in the next few years, several of which are designed specifically with the intent of improving estimates of the standing stock of carbon in biomass, and changes in those stocks through time. The UNFCCC process would benefit from refinement and application of these approaches and from improved data in developing policies designed to reduce emissions from deforestation and forest degradation.

## Competing interests

The authors declare that they have no competing interests.

## Authors' contributions

SJG conceived the review and drafted the majority of the manuscript. NTL, AB and MS conducted the analyses summarized in the Figure and Table. TJ contributed the section on good practice guidance. SJG, WW, JK summarized of types of satellite measurements. RH and the other authors read, edited, and approved the final manuscript.
